# Social work support and unmet social needs in life after stroke: a cross-sectional exploratory study

**DOI:** 10.1186/s12883-019-1451-y

**Published:** 2019-09-06

**Authors:** Sophie Lehnerer, Benjamin Hotter, Inken Padberg, Petra Knispel, Dike Remstedt, Andrea Liebenau, Ulrike Grittner, Ian Wellwood, Andreas Meisel, on behalf of the BSA Long Term Care Study Group

**Affiliations:** 1 Center for Stroke Research Berlin, Charité University Medicine Berlin, Charitéplatz 1, 10117 Berlin, Germany; 2 Department of Neurology, Charité University Medicine Berlin, Charitéplatz 1, 10117 Berlin, Germany; 3 NeuroCure Clinical Research Center, Charité University Medicine Berlin, Charitéplatz 1, 10117 Berlin, Germany; 4 Clinical Epidemiology and Health Services in Stroke, Charité University Medicine Berlin, Charitéplatz 1, 10117 Berlin, Germany; 5 Berlin Stroke Alliance (BSA), Charitéplatz 1, 10117 Berlin, Germany; 6 Institute of Biometry and Clinical Epidemiology, Charité University Medicine Berlin, Charitéplatz 1, 10117 Berlin, Germany; 7 Berlin Institute of Health (BIH), Anna-Louisa-Karsch 2, 10178 Berlin, Germany; 8 Department of Public Health and Primary Care, Cambridge Institute of Public Health, University of Cambridge, Cambridge, CB2 0SR UK

**Keywords:** Stroke, Long-term care, Social situation, Unmet social needs, Assessment tools, Screening social work

## Abstract

**Background:**

Stroke patients are often affected by long-term disabilities with needs concerning social issues. There is relatively little consideration of social recovery of patients and the support required to return to work, receive social benefits, participate in daily life activities, maintain contact with family and friends and to organize financial affairs. In our study we aimed to investigate if existing tools record social needs adequately. We analyzed the current provision of social support provided in long-term care after stroke and whether unmet social needs were associated with quality of life, caregiver burden, overall function and degree of disability.

**Methods:**

Our analysis is part of the Managing Aftercare of Stroke study (MAS-I), a cross-sectional exploratory study of patient needs 2–3 years after initial stroke. Assessment tools included the Nikolaus-score (social situation), the EuroQoL (quality of life), the German Burden Scale for Family Caregivers (caregiver burden), the modified Rankin Scale (disability / dependence), Stroke Impact Scale (function and degree of disability) and the Stroke Survivor Needs Questionnaire (unmet needs).

**Results:**

Overall 57 patients were included in MAS-I, with ten patients classified in urgent need of socio-economic support according to the Nikolaus-score. Patients with lower than normal Nikolaus-score had a higher degree of disability. Thirty percent of all patients had never received professional social support. Social worker contact happened mostly during the stay in acute hospital or rehabilitation institution. Only four patients (11%) reported long-term support after discharge. Apart from social worker contact during acute care, 43% of patients had unmet needs in the long-term aftercare. Forty percent of all patients included in MAS-I were recommended for social work intervention after an in-depth analysis of their situation. Finally, we saw that unmet social needs were associated with lower quality of life and higher caregiver burden.

**Conclusions:**

Our data suggest significant unmet needs in social care in long-term stroke patients. Screening tools for unmet social needs such as the Nikolaus-score do not holistically report patients’ needs.

**Trial registration:**

Clinicaltrials.Gov NCT02320994. Registered 19 December 2014 (retrospectively registered).

**Electronic supplementary material:**

The online version of this article (10.1186/s12883-019-1451-y) contains supplementary material, which is available to authorized users.

## Background

Stroke patients are often affected by long-term disabilities with complex needs requiring a combination of medical, nursing, therapeutic and social interventions to address a wide range of concurrent symptoms such as paresis, spasticity, pain, aphasia, cognitive impairment and depression [1–3]. Several approaches have been proposed to provide comprehensive assistance to patients after stroke [4, 5], however, many focus on promoting early return to the community rather than on-going care [6–13].

For post-stroke impairments like sensory-motor dysfunction or aphasia as well as for post-stroke comorbidities such as spasticity, pain, cognitive impairment and depression, clinical guidelines [10] and some therapeutic treatment options are available [14–20]. On the other hand, there is relatively little attention to the social recovery of patients. Support for social recovery may include professional support to return to work, access social benefits, participate in activities of daily life, maintain contact with family and friends and organize financial affairs [21–23]. According to the “Burden of stroke in Europe” report there is a lack of long-term support (that we term “aftercare”) for stroke in every European country [24]. There are some studies focussing on specific issues or patients’ experiences [25], but few have aimed to achieve a holistic view of the social situation in long-term aftercare of stroke patients [22] and its interdependency with post-stroke sequelae.

Here we aimed to investigate if existing tools like the Nikolaus-score [26] and the Stroke Survivor Needs questionnaire [27] record unmet social needs adequately, and what kind of additional information is relevant. This includes unmet needs which are likely to occur, but which cannot be addressed at time of assessment.

Furthermore, we aimed to evaluate the current provision of social support provided in long-term care after stroke and the prevalence of unmet social needs in the long-term and whether these unmet needs were associated with quality of life (EuroQoL (EQ-5D-3 L) [28]), the caregiver burden (Häusliche Pflegeskala, HPS-k [29]), the overall function and degree of disability (modified Rankin Scale (mRS) [30] and the Stroke Impact Scale [31].

## Methods

### Study-design

The present exploratory analysis is part of the MAS-I-study, a cross-sectional exploratory study of post rehabilitation patient needs and caregiver burden after stroke [11]. The dataset represents a first step in a more complex project, aiming to gather information on unmet medical and social needs in the long-term aftercare for stroke in the outpatient setting. Briefly, stroke patients from two previous acute clinical studies were invited 2–3 years after the initial event to attend the outpatient department for a comprehensive interview and examination carried out by a trained neurologist and social worker using validated standard measures of self-reported needs, quality of life, overall outcome, spasticity, pain, aphasia, cognition, depression, secondary prevention, social needs and caregiver burden [11]. No financial incentive was provided, but transport was organized and paid for, if necessary. Written consent was given before participation; details are provided in the section “Declarations”. The study received ethics committee and data protection approval by the institutional review board of Charité University Medicine, Berlin (reference EA1/183/14) and was registered on clinicaltrials.gov (NCT02320994).

### Scores

In the current study we explored the patients’ contact with social workers, assessed by self-reported previous support and its association with the social situation assessed with the German Nikolaus-score [26] (unmet social needs are indicated by sum score values of 17 or below, ranges from 0 to 25 (no unmet social needs), see Additional file 2 for an unofficial translation by the authors). Furthermore, we explored the quality of life assessed with the EuroQoL (EQ-5D-3 L, index ranges from 0 (poor quality of life) to 1) [28], the caregiver burden with the German Burden Scale for Family Caregivers (Häusliche Pflegeskala, HPS-k, ranges from 0 to 30 (high caregiver burden)) [29], the overall function and degree of disability (modified Rankin Scale (mRS), ranges from 0 (no symptoms) to 6 (death)) [30] and one item (item 8 - participation in activities of daily living) from the Stroke Impact Scale [31]. To explore the scope and content of the tools we also assessed the patients using components of the Stroke Survivor Needs questionnaire [27]. This questionnaire does not work as a score, but contains additional information not provided by the Nikolaus-score, with fourteen questions inquiring about unmet social needs like need for help in the household and personal care, in managing finances and applying for social benefits (listing of the fourteen items and dividing into seven domains see Table 1). Furthermore, the validated scales were integrated in the interview and conversation between social worker and patient. We recorded standardized individual recommendations issued by a stroke neurologist and social worker after an in-depth analysis of the current care according to national and international clinical guidelines [10]. We used these recommendations as a surrogate measure of current gaps in the ongoing care of these patients. If unmet social needs were identified, the patient received a recommendation for further social work intervention which was performed in the stroke service point of the Berlin Stroke Alliance [32]. This intervention however, did not form part of the study. Recommended social work interventions address non-medical needs in primary health care [33]. This includes access to out-patient nursing care (including general home care, palliative care, family care and short term home care), home adaptation and aids (including changes around the house, emergency house calls and other aids at home), help with mobility and transport (including driving services for recreation or provision of companions for people with decreased mobility). Furthermore social work intervention implies the provision of information about out-patient and in-patient rehabilitation and help with applications for benefits (specific to the German health care system) [34].

Relevant licences were obtained for scores which were not available licence-free.

### Statistical analysis

Statistics were calculated using SPSS 24.0 software (IBM, Armonk, NY). Standard descriptive statistics were chosen depending on the scaling and distribution of the variables. Associations between the assessments were calculated using Pearson’s χ^2^ and Mann-Whitney U depending on the scaling of the variables as specified in detail in the result Tables. A two-sided significance level of α = 0.05 was considered. No adjustment for multiple testing was applied in this exploratory study.

## Results

### Unmet social needs and recommendation of social work intervention

Overall 57 patients were included in MAS-I [11]. As Table 1 shows, all patients were assessed for social needs using the Nikolaus-score with 10 patients needing socio-economic support according to the score results. Of these patients, nine received the recommendation to be supported by a social work intervention due to low Nikolaus-score. Another twelve patients, although with Nikolaus-score values higher than the cut-off value of 17 points, were referred to a social worker intervention. This is because the recommendations for social work intervention made by the clinician were not only based on the Nikolaus-score, but also on the interview by the social worker that included questions on the stroke survivor’s needs. The additional information provided by the patient while answering the questionnaire and the informal check of the social worker whether preconditions for social benefits were given, were the trigger to recommend social work intervention. The above mentioned 12 patients in Table 1 expressed unmet needs in the domain “information about application for social benefits, managing finances and reemployment required”. Twenty-four patients were not referred to a social worker, although they showed needs in the questions from the Stroke Survivor Needs questionnaire. These needs are not currently covered under the legal entitlements in Germany, unless individual preconditions have been met and some elements of care may not be covered by nursing care or personal care insurance.

**Table 1:** Unmet social needs (Nikolaus-score), recommendation of social work intervention and in-detail analyses of the Stroke Survivor Nneeds questionnaire

ID (patients)	Unmet social needs (< 17 points in the Nikolaus-score)	Social work intervention recommended	Number of unmet needs in defined items of the Stroke Survivor Needs questionnaire (domain^1^)
1	yes	yes	6 (A,2xB, C, D, F)
2	yes	yes	1 (C)
3	yes	yes	4 (A, B, D)
4	yes	yes	2 (A, C)
5	yes	yes	6 (A, 2xC, D, 2xG)
6	yes	yes	2 (F, G)
7	yes	yes	3 (D, F, G)
8	yes	yes	2 (D, F)
9	yes	yes	0
10	yes	Data missing	2 (C, F)
11	no	yes	2 (2xC)
12	no	yes	1 (D)
13	no	yes	1 (A)
14	no	yes	4 (A, C, F, G)
15	no	yes	4 (2xC, F, G)
16	no	yes	1 (D)
17	no	yes	0
18	no	yes	3 (A, 2xC)
19	no	yes	2 (A, E)
20	no	yes	2 (A, F)
21	no	yes	1 (G)
22	no	yes	2 (F, G)
23	no	Data missing	1 (A)
24	no	Data missing	2 (C, F)
25	no	Data missing	0
26	no	no	1 (F)
27	no	no	1 (A)
28	no	no	2 (2xC)
29	no	no	1 (A)
30	no	no	1 (B)
31	no	no	4 (A, 2xC, E)
32	no	no	1 (F)
33	no	no	3 (A, C, F)
34	no	no	1 (G)
35	no	no	1 (F)
36	no	no	1 (F)
37	no	no	2 (C, F)
38	no	no	1 (A)
39	no	no	3 (2xD, F)
40	no	no	3 (A, C, F)
41	no	no	1 (A)
42	no	no	1 (E)
43	no	no	1 (A)
44	no	no	1(A)
45	no	no	2 (C, F)
46	no	no	3 (2xC, F)
47	no	no	2 (A, B)
48	no	no	4 (A, B, D, E)
49	no	no	1 (A)
50	no	no	2 (A, D)
51	no	no	0
52	no	no	0
53	no	no	0
54	no	no	0
55	no	no	0
56	no	no	0
57	no	no	0

^1^Domain (Number of item in the stroke survivor needs questionnaire [27])

A = more information about stroke required [1]

B = personal care or professional help for household required [17, 18]

C = further equipment, adaptions outside the home or moving home required [19–21]

D = advice about driving after stroke or travelling with public transport required [23, 24]

E = advice about physical relationships with partner required [29]

F = access to a support group required [32]

G = information about application for social benefits, managing finances and re-employment required [36–39]

### Characteristics of patients with unmet social needs

While age and sex (see Table 2) was similarly distributed in patients with values above and below 17 points in the Nikolaus-score (median 19), the degree of disability and dependence in daily activities according to the modified Rankin Scale was higher in patients with less than 17 points in the Nikolaus-score (median 3 vs. 2, *p* = 0.018). Patients with low Nikolaus-score (< 17 points) furthermore reported a more accentuated negative impact of stroke on their life (Stroke Impact Scale, median 21 vs. 34, *p* = 0.002), and a lower quality of life (EQ-5D-3 L index value, 0.70 vs. 0.89, *p* = 0.02) (all see Table 2). In addition, the nearest relatives of the patients with low Nikolaus-scores (< 17 points) reported a higher caregiver burden (HPS-k, median 21 vs. 6, *p* = 0.053, Table 2) compared to caregivers of patients with higher Nikolaus-scores. Nikolaus-score was positively associated with net income (Spearman r = 0.39, *p* = 0.008), with a mean net income of €1300 Euro in patients below 17 points versus €2225 Euro in patients with higher score values in the Nikolaus-score (Table 2), signifying more unmet social needs in patients with a higher economic strain.

**Table 2:** Characteristics of patients with unmet social needs (Nikolaus-score)

	Total (*n* = 57)	Urgent need of social help (Nikolaus-score < 17) (*n* = 10)	No urgent need of social help (Nikolaus-score > 16) (*n* = 47)	*p*-value
Age, mean (SD)	70 (10) (*n* = 55)	68 (12) (n = 10)	71 (9) (n = 45)	0.623 ^a^
Male, n (%)	33 (58%) (*n* = 57)	4 (40%) (*n* = 10)	29 (62%) (n = 47)	0.207 ^b^
*Scores at MAS visit*				
Nikolaus-score median (IQR)	19 (17–22) (n = 57)	14 (12–16) (n = 10)	20 (19–22) (*n* = 47)	
Modified Rankin Scale (mRS) median (IQR)	2 (1–3) (n = 57)	3 (2–4) (n = 10)	2 (1–3) (n = 47)	0.018 ^a^
Stroke-Impact-Scale median (IQR)	32 (23–40) (n = 57)	21 (18–25) (n = 10)	34 (26–40) (n = 47)	0.002 ^a^
EQ-5D-5 L-Index median (IQR)	0.81 (0.70–1.00) (n = 57)	0.70 (0.38–0.79) (n = 10)	0.89 (0.70–1.00) (n = 47)	0.020 ^a^
Caregiver burden scale (HPS-k) median (IQR)	6 (1–11) (*n* = 24^c^)	21 (values: 6; 27) (n = 3)	6 (1–9) (*n* = 21)	0.053 ^a^
Years of education Median (IQR)	14 (12–17) (n = 56)	14 (12–17) (n = 10)	14 (12–17) (*n* = 46)	0.957 ^a^
Net income in Euro median (IQR)	2200 (1200–2500) (n = 47)	1300 (516–2500) (*n* = 9)	2225 (1425–2550) (*n* = 38)	0.170 ^a^

^a^Mann-Whitney-U Test, ^b^ Chi-Square Test, ^c^ only 24 patients had family members, who were caregivers and consented participation at the study

Accordingly, patients receiving a recommendation for intervention by a social worker were more severely affected by their stroke (median mRS 3 vs. 1, *p* = 0.13, median Stroke-Impact-Scale 24 vs. 34, *p* = 0.014, Table 3). Thus, more severely affected patients (mRS > 2) had a lower Nikolaus-score (median 19 vs. 21, *p* = 0.013) and more often received recommendations for social work interventions (57% vs. 28%, *p* = 0.035, Table 4) compared to less affected patients.

**Table 3:** Characteristics of patients with recommendation for social work intervention (*n* = 53)

Social work intervention recommended:	NO (*n* = 32)	YES (*n* = 21)	*p*-values
Age (mean (SD))	71 (9) (*n* = 30)	69 (11) (n = 21)	0.443 ^a^
Male, n (%)	16 (50%) (n = 32)	13 (62%) (n = 21)	0.394 ^b^
Modified Rankin Scale (mRS) median (IQR)	1 (1–3) (n = 32)	3 (2–3) (n = 21)	0.013 ^a^
Nikolaus-score median (IQR)	21 (19–22) (n = 32)	17 (15–21) (n = 21)	0.005 ^a^
Stroke-Impact-Scale median (IQR)	34 (27–40) (n = 32)	24 (21–33) (n = 21)	0.014 ^a^
EQ-5D-5 L-Index median (IQR)	0.89 (0.79–1.0) (n = 32)	0.79 (0.70–0.89) (n = 21)	0.060 ^a^

^a^Mann-Whitney-U Test, ^b^ Chi-Square Test

**Table 4:** Modified Rankin Scale (mRS): Unmet needs (Nikolaus-Score) and recommendation for social work intervention (n = 57)

	mRS < 3 (low-moderate degree of disability) (*n* = 34)	mRS > 2 (severe degree of disability) (*n* = 23)	*p*-value
Age (mean (SD))	71 (9) (*n* = 33)	69 (11) (n = 22)	0.491 ^a^
Male, n (%)	22 (65%) (n = 33)	11 (48%) (n = 23)	0.205 ^b^
Nikolaus-score median (IQR)	21 (19–22) (n = 34)	19 (15–20) (n = 23)	0.013 ^a^
Social work intervention recommended (n = 21/53), n (%)	9 (28%) (n = 32)	12 (57%) (n = 21)	0.035 ^b^

^a^Mann-Whitney-U Test, ^b^ Chi-Square Test

### Contact with social workers in the aftercare of stroke

During the interviews, all patients were questioned about previous contact with social workers. Table 5 shows, that seventeen patients (30%) had never received professional social support. If social support was provided, the consultation happened mostly during the stay in acute hospital or neuro-rehabilitation institution (37 of 39 patients, see Additional file 1). Only 11% (*n* = 4/36) reported long-term support after discharge, and 61% (*n* = 22/36) have not been informed in-detail about their situation and perspectives (for missings, detailed content and quality of social support see Table 6). Patients who received neuro-rehabilitation treatment (*n* = 45/55) were more likely to be contacted by a social worker than patients without neuro-rehabilitation treatment (35/45 [78%] vs. 1/10 [10%], *p* < 0.001, additional results without table).

**Table 5:** Contact with social workers (n = 57)

	Never had contact with social worker (*n* = 17)	Had contact with social worker (*n* = 40)	*p*-value
Age (mean (SD))	71 (12) (n = 17)	70 (9) (n = 38)	0.247 ^a^
Male, n (%)	12 (70%) (n = 17)	21 (53%) (n = 40)	0.251^b^
Modified Rankin Scale (mRS) median (IQR)	1 (1–3) (n = 17)	2 (1–3) (n = 40)	0.058 ^a^
Years of education Median (IQR)	16 (12–19) (n = 17)	14 (12–16) (*n* = 39)	0.352 ^a^
Net income in Euro median (IQR)	2250 (1300–2500) (*n* = 15)	2050 (913–2650) (n = 32)	0.714 ^a^
Nikolaus-score median (IQR)	21 (17–22) (n = 17)	20 (17–21) (n = 40)	0.313 ^a^
Nikolaus-score < 17 Points n (%)	3 (18%) (n = 17)	7 (18%) (n = 40)	0.989 ^b^
Social work intervention recommended n (%)	5 (31%) (*n* = 16)	16 (43%) (*n* = 37)	0.412 ^b^

^a^Mann-Whitney-U Test, ^b^ Chi-Square Test

**Table 6:** Content and quality of social support

	n (%)	Total n
Provided with detailed information about the situation	14 (39%)	36
Provided with a leaflet/flyer or brief information material	20 (54%)	37
Information about items such as rehabilitation, nursery care, benefits and social rights	9 (25%)	36
Help to fill out application forms	12 (33%)	36
Long-term support after discharge	4 (11%)	36

Patients who received social support were more severely affected by stroke than patients not receiving social support (median mRS 2 vs. 1, *p* = 0.058, Table 5). In 16 out of 37 (43%) patients who had contact with social workers, further social work intervention was recommended during the stay in the acute hospital or rehabilitation center due to unmet social needs. Table 5 shows that average age, sex distribution, years of education and net income were similar in patients receiving social support or not. We did not see pronounced differences in the prevalence of unmet needs between patients with or without previous contact with social workers.

## Discussion

One third of all patients included in the MAS-I study reported having had no contact with social workers. Possibly, contact with social workers in the acute phase of stroke was not always recognized, perhaps because of being overwhelmed with information or impaired cognition [35]. Stroke patients are prone to reporting bias concerning services received [36]. Ten patients of the presented study, who received rehabilitation, reported no contact with social workers. In Germany, application for rehabilitation is usually connected to a social work intervention, so these ten patients were not aware of the social support they received. On the other hand, they may not have understood this support as social support, and would perhaps have required more support in other domains e.g. preparation for their return to home. Therefore, the social work service - especially in the acute - phase should be adapted to the patient’s situation. This would mean a proper introduction of the consulting social worker, enough time and adapting the information load to the patient’s cognitive abilities.

The data demonstrate that most of the social worker contact takes place during the stay in hospital or rehabilitation. Unfortunately, our questionnaire did not differ between social support in hospital and rehabilitation. In the acute phase, hospital social support usually assists in applying for rehabilitation. In the second phase, social support assists in discharge, aiming to organize nursing services for severely affected patients. For this reason, less affected patients did not receive as much social consultation. Even if there was no need for rehabilitation or nursing services, unmet social needs remain; e.g. getting advice on how to obtain benefits, information on driving after stroke, contact details of medical doctors and self-help groups, help with arranging housekeeping and dealing with the disability in the context of relationships and sexuality [34, 37–39]. Patients need social work services to begin early in acute treatment and continue after discharge [40]. Our data indicates that unfortunately there was scarce social support after discharge in the long-term. Services like the outpatient stroke service point in Berlin [34] may provide a model to address this problem. The role of social workers is crucial, serving to liaise between different institutions in order to assist patients. In our opinion, this role does not receive adequate recognition. More research in this field would emphasize the importance of post-acute stroke services [41]. Evaluating the effect of social worker interventions is part of our consecutive MAS-II study (Managing Aftercare for Stroke - A Longitudinal Complex-interventional Study in Post-rehabilitation Stroke Patients, clinicaltrials.gov NCT03097146).

Our data suggests that previous social worker contact does not reduce social needs in the aftercare (described by the patients themselves as well as evaluated by the neurologist). This might be because initial social worker contact is mainly in hospital, and rehabilitation takes place to organize rehabilitation and nursing home care in more severely affected patients. Independent of the severity of their stroke, patients in the long-term have unmet (or yet to be identified) social needs that have not been covered by their previous contact with a social worker. This emphasizes that in the years after stroke, health care providers should regularly screen for unmet social needs. Over 50% of patients and their relatives visit social services > 6 months after the initial event. This takes place when patients have returned home or to a nursing home, adaption to this new everyday life has happened and unmet needs are discovered (Cameron & Gignac, 2008) [34]. Our data demonstrates that patients with unmet needs evaluated by the Nikolaus-Score in the long-term were more severely affected and had a lower income. Furthermore, they have a lower quality of life and a higher caregiver burden. Caregivers often experience stress and poor mental and physical health [42], which can lead to poor rehabilitation outcome of stroke survivors [43, 44].

In the MAS-I study several patients received a recommendation for social work intervention by the stroke neurologist, but were not classified by the Nikolaus-score as being in need of social help. In terms of methods, an internationally validated screening tool for social needs after stroke is still required. The Nikolaus-score is widely used in Germany, but does not seem to be an appropriate up-to-date tool. First, it is not specifically validated for stroke patients, but for a general geriatric population [26]. Second, the items regarding the housing situation are outdated: warm water, heating, toilets or telephone sockets are basic standards nowadays, but are weighted as important items in the score, falsely increasing the score. Third, the Nikolaus-score combines items like “apartment on one floor, spacious and wheelchair accessible” which aggregates complex accessibility issues. Fourth, these items might not be relevant if the stroke patient is not wheelchair bound, but the score-point counts nevertheless. The recommendations for social work intervention made by the clinician included the results of the Nikolaus-score but also took into consideration information given by the patient during the interview, which our social worker had not asked about in the standardized questionnaires. Furthermore, we considered 14 of the 40 items of the Stroke Survivor Needs questionnaire, which were seen as relevant for addressing social issues. Twenty-four patients were not referred to a social worker although showing needs in these components of the Stroke Survivor Needs questionnaire. These needs are not currently covered under the legal entitlements in Germany, unless individual preconditions have been fulfilled and some elements of care may not be covered by personal care insurance.

It would be desirable to develop an evaluation tool for social needs considering personal contextual factors of the ICF (International Classification of Functioning). This includes relevant personal factors to describe the background of an individual’s life and living [45]. Moreover, such a questionnaire should focus on contemporary topics relevant for stroke patients, such as inpatient and outpatient rehabilitation, medical treatment, medical rehabilitation, therapeutic or preventive services, questions around medical and personal care insurance, social legislation, pensions and disability benefits and information on self-help groups [34]. Not only should the unmet needs be evaluated, but also the legal preconditions based on which social benefits can be applied for. A literature review shows that longer-term problems of stroke patients concern social and emotional consequences [25, 46]. Even mildly affected patients seek psychological support as anxiety after stroke is common [47, 48]. The emotional as well as the social situation should be recorded since multiple studies reveal an association between depression and low-social support [49].

Relatives of stroke patients play an essential role in providing care. It is important to include the caregivers` situation in the evaluation. So far there is no standardized procedure in Germany of when to provide support to caregivers concerning follow-up care for patients [50], even though many suffer from psychological and physical stress [51]. Two-thirds of the clients contacting stroke service points in Berlin are caregivers [34]; this reveals the need to involve the nearest relative in evaluation and support. In the MAS-I study 24% of the caregivers reported moderate to high levels of stroke-related caregiver burden [11].

Several approaches have been suggested to close the gap between inpatient and outpatient care [52–54], however, social care has been understudied. An effective primary care-based stroke aftercare service must have a broad focus and must be based on an individual record of unmet needs including social needs [55]. Thus far, in the out-patient setting it is difficult to record unmet needs after stroke appropriately. Similar levels of impairment can impact individuals differently, depending on the context. Also the individual resources like personality and caregiver’s support to deal with long-term complications are very different [56] and can lead to comorbidities like depression as well as a low quality of life [57]. Patients who receive little information about their situation are more likely to be depressed [58]. Low socioeconomic status increases the risk for stroke [58]. Socially isolated stroke patients are more likely to have recurrent stroke and have a higher mortality [59]. Social needs require to be identified to be treated.

Due to a rather small sample size and the fact that severely disabled patients and their carers were less likely to attend, we advise caution when interpreting these findings. On the other hand, a strength of the studied population is its detailed characterization, which allows for exploratory analysis.

## Conclusions

In the present study we see that only two thirds of stroke patients have contact with social workers. This contact mainly takes place during the in-patient setting. Although they may have had social worker contact previously, patients continue to have unmet needs for long-term aftercare. Forty percent of all patients included in the MAS-I study received the recommendation for social work intervention after an in-depth analysis of their situation. Our data suggest that screening tools for unmet social needs as the Nikolaus-score are not appropriate to report the needs holistically. Finally, we saw that unmet social needs were associated with lower quality of life and higher care giver burden.

The findings warrant large prospective, longitudinal studies identifying and validating screening tools for unmet social needs and to develop comprehensive management of unmet social needs to improve medical and social outcomes in stroke.

## Additional files

Additional file 1: Cross table: Prevalence and time of contact with social worker (*n* = 56, 1 missing). This file shows in a cross table the numbers and percentages of patients who have or have not had contact with social workers during and/or after stay in hospital or rehabilitation. The McNemar Test shows a significant result (*p* < 0.001). (DOCX 13 kb)

Additional file 2: English version of the Nikolaus Score [26] translated by the authors. We explicitly declare that this is neither an official nor a verified translation. (DOCX 20 kb)

## Data Availability

The datasets used and/or analysed during the current study are available from the corresponding author on reasonable request.

## Abbreviations

HPS-k: Häusliche Pflegeskala
ICF: International Classification of Functioning
IQR: Interquartile range
MAS: Managing aftercare of stroke (study)
mRS: modified Rankin Scale
n: number
