# Supplementary material for: Social work support and unmet social needs in life after stroke: a cross-sectional exploratory study
Source: BMC Neurol. 2019 Sep 6;19:220. doi: 10.1186/s12883-019-1451-y (PMC6729017; doi:10.1186/s12883-019-1451-y)
Supplement: Supplementary file 1 — Cross table: Prevalence and time of contact with social worker (n = 56, 1 missing). This file shows in a cross table the numbers and percentages of patients who have or have not had contact with social workers during and/or after stay in hospital or rehabilitation. The McNemar Test shows a significant result (p < 0.001). (DOCX 13 kb) [file 12883_2019_1451_MOESM1_ESM.docx]

**Additional file 1: Cross table: Prevalence and time of contact to social worker (n=56, 1 missing)**

|  | **After** stay in hospital or rehabilitation **no** contact to social worker | **After** stay in hospital or rehabilitation contact to social worker |  |
| --- | --- | --- | --- |
| **During** stay in hospital or rehabilitation **no** contact to social worker | 17 (30.4%) | 2 (3.6%) | 19 (33.9%) |
| **During** stay in hospital or rehabilitation contact to social worker | 33 (58.9%) | 4 (7.1%) | 37 (66.1%) |
|  | 50 (89.3%) | 6 (10.7%) | 56 (100.0%) |

McNemar Test: p<0.001
