# Supplementary material for: Social work support and unmet social needs in life after stroke: a cross-sectional exploratory study
Source: BMC Neurol. 2019 Sep 6;19:220. doi: 10.1186/s12883-019-1451-y (PMC6729017; doi:10.1186/s12883-019-1451-y)
Supplement: Supplementary file 2 — English version of the Nikolaus Score [26] translated by the authors. We explicitly declare that this is neither an official nor a verified translation. (DOCX 20 kb) [file 12883_2019_1451_MOESM2_ESM.docx]

Additionial file 2

English version of the Nikolaus Score^1^ translated by the authors. We explicitly declare that this is neither an official nor a verified translation.

Part 1: Social contacts and support

1. How do you live?

- Alone for a long time 1
- Recently alone (< 1 year) 0
- With family members or with a sprightly partner 1
- With a life partner who needs help them self 0

1. Do you have people (including professional helpers) who you can rely on and who can help you regularly at home? (give the number of people)

………………………..

- Reference person present 1
- No reference person present (continue with question 5) 0

1. How often do you see this person/ these persons?

- Several times a day/ daily 1
- Once a week 1
- Rarely (One to two times a month) 0
- (Almost) never 0

1. What is your relationship with the above-mentioned person(s)?

- Relationship is harmonious and trusting 1
- Relationship is partially conflict-laden and tense 0

1. How have your contacts developed recently?

- Have gained new acquaintances 1
- No change 1
- Some contacts I had to give up 0
- Have lost almost all important contacts (e.g. life partner deceased) 0

1. Are you satisfied with this condition?

- Feel well taken care of all around 1
- It’s how it goes, have to be satisfied 0
- Feeling lonely and abandoned 1

Subtotal (contacts) ………………………………………………………………………………………………………….

Part 2: Social activities

1. What was your profession? …………………………………………………………
2. What hobbies (handicrafts, making music, gardening, collecting stamps, etc.) or interests (lectures, excursions, theatre, sports, reading books, going to church, meeting senior citizens, keeping grandchildren, etc.) do you have that you still regularly pursue? (how many?)

………………………………………………………

Hobbies/ interests present 1

No hobbies/ interests 0

1. Do you have a pet?

Yes 1

No 0

1. How often do you leave your apartment? (Shopping, transactions, walking, (doctor) visits, gardening, etc.)

- Daily 1
- At least once to twice a week 1
- Less than once a week 0
- (Almost) never 0

1. How have your interests developed recently?

- Still have new plans and interests 1
- Unchanged 1
- Had to give up some interests 0
- Have lost almost all interests 0

1. Are you satisfied with this condition?

- All the way, I don't feel affected 1
- Feel restricted already, must be satisfied 0
- No, I am severely handicapped by age/illness 0

Subtotal (activities) …………………………………………………………………………………………………………

Part 3: Housing situation

1. Stairs

- Apartment on the ground floor or lift in the house 1
- Many stairs, first floor or higher 0

1. Comfort

- Apartment on one floor, spacious and wheelchair accessible 1
- Narrow conditions, door thresholds, many carpets 0
- Several living levels, not wheelchair accessible 0

1. Heating

- Good and convenient heating (oil or gas central heating) 1
- Badly heated and hard work to keep heated (coal or oil furnaces) 0

1. Water

- Warm water in kitchen and/or bath 1
- No hot water available 0

1. Bathroom

- Inside the apartment, wheelchair accessible 1
- Small, not wheelchair accessible, outside the apartment 0

1. Telefone

- Available 1
- Not Available 0

1. Lighting

- Staircase and corridors bright, enough light switches 1
- Staircase and corridors dimly lit 0
- Few light switches 0

1. Shopping

- All shops for daily needs are easily accessible 1
- Only bakers/butchers nearby 0
- All shops further away 0

1. Local traffic

- Bus stop etc. nearby (< 1km) 1
- Next stop further away 0

1. Dwelling

- Lived in the apartment for a long time (>5 years) 1
- Moved into an apartment within the last 5 years 0

1. Do you feel comfortable in your apartment and the residential area?

- I am very satisfied with the living situation 1
- It’s how it goes, must be satisfied 0
- I am unhappy 0

Subtotal (housing) …………………………………………………………………………………………………………

Part 4: Economic conditions

1. How much money is at your disposal each month?

…………………………………………

1. Do you make ends meet with your money?

- Yes 1
- Only a little 0
- No 0

1. Do you have savings, assets (own house)? (how much?)

- Yes, sufficient 1
- Only a little 0
- No 0

1. Do you manage your finances yourself?

- Yes 1
- No 0

Subtotal (economy) ………………………………………………………………………………………………………..

The total number of points is calculated from this:

Subtotal (contacts) …………………….

+ Subtotal (activities) …………………..

+ Subtotal (housing) …………………...

+ Subtotal (economy) …………………..

Total amount: ……………………………………………………

1) Nikolaus T, Specht-Leible N, Bach M, Oster P, Schlierf G. Social aspects in diagnosis and therapy of very elderly patients. Initial experiences with a newly developed questionnaire within the scope of geriatric assessment (Article in German). Z Gerontol. 1994,. 27(4):240-5.,
